# The new coronavirus and the risk to children's health

**DOI:** 10.1590/1518-8345.0000.3320

**Published:** 2020-04-22

**Authors:** José Manuel da Silva Vilelas

**Affiliations:** 1 Escola Superior de Saúde da Cruz Vermelha Portuguesa, Lisboa, Portugal.


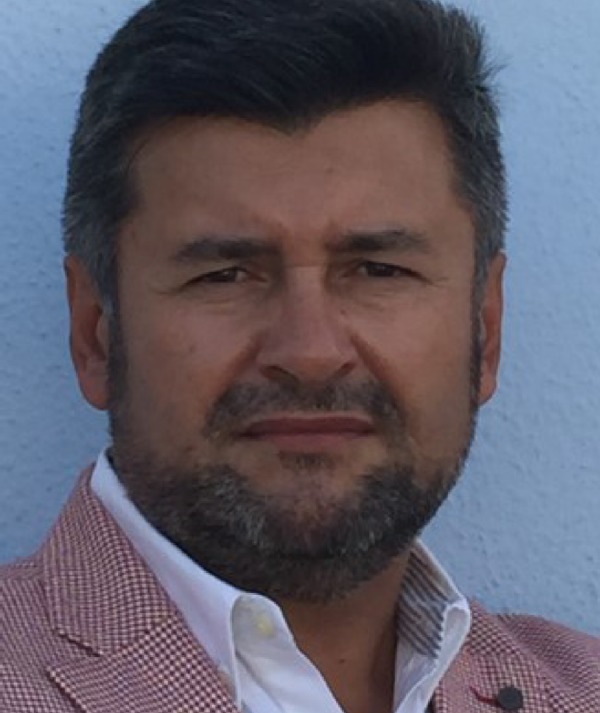


Since December 2019, an epidemic caused by the severe acute respiratory syndrome coronavirus 2 (SARS-CoV-2) occurred unexpectedly in Wuhan, Hubei province, China and quickly spread from Wuhan to other areas of China and other countries. The World Health Organization named this new coronavirus disease COVID-19, resulting from the combination of the words Corona, Viruses and Disease, with an indication of the year 2019, the year of its appearance. The General Directorate of Health of Portugal states that the transmission of COVID-19 can occur through respiratory droplets, direct contact with respiratory secretions, infected feces or contaminated surfaces and by air, through aerosol generators^(^ 1 ^)^.

There are relatively few reported cases of COVID-19 infection in children, compared to the total number of cases in the general population. In February 2020, 2.4% of the 75,465 cases in China (confirmed and suspected) occurred in children, as well as in Italy with 1.2%^(^ 2 ^)^ and 5% in the United States of America^(^ 3 ^)^. Data from the General Directorate of Health of Portugal, from March 29, 2020, show that 1% of children under 10 years old and 2.3% of adolescents between 11 and 19 years old were presenting COVID-19. At the moment, there are no cases of death in this age group^(^ 1 ^)^. One of the explanations for this disease not being prevalent in children may be because they are less exposed to the virus and have less indications for testing for SARS CoV-2 because, in most cases, they have mild symptoms similar to those of a common flu. The function of innate immunity to respiratory tract infection is greater in children than in adults, because the adaptive immune response in children is superior and the protein that binds to the angiotensin-converting enzyme is less mature in younger people, which makes such binding difficult. Thus, children's ability to trigger an acute inflammatory response to SARS-CoV-2 is weak, which can also contribute to a better outcome. Such particularities, however, do not eliminate the possibility of serious cases and even death, especially in children with comorbidities^(^ 4 ^)^.

Regarding mother-fetus intrauterine vertical transmission, there is still no scientific evidence to demonstrate its existence. COVID-19 was also not detected in breast milk^(^ 5 ^)^. However, the main concern is whether an infected mother can transmit the virus through respiratory droplets. Thus, breastfeeding during maternal COVID-19 infection is not contraindicated by the Centers for Disease Control and Prevention and the Royal College of Obstetricians and Gynaecologists, but precautions must be taken to prevent the spread of the virus to the newborn, including washing your hands before touching it and wearing a face mask. In the case of breast milk extraction, the recommendations for cleaning the breast pumps after each use must be strictly observed^(^ 6 ^)^.

Although the immediate risk of COVID-19 in children is low, it is important to monitor the situation and its evolution. At this stage, the concern about COVID-19 can make children and their families anxious. Several countries have implemented social confinement and distancing, which means maintaining a safe distance (approximately one meter) from others and avoiding meeting spaces with more than five people. In case of confinement at home, parents are often the best and closest resource for their children to seek help. Games and play can be strategies for distraction and communication with children. Toys should be cleaned and disinfected with soap and water, a disinfectant or sodium hypochlorite solution (10 ml/1 liter of water). This virus is inactivated after five minutes^(^ 7 ^)^.

The current outbreak of COVID-19 remains serious worldwide and has been designated as a Public Health emergency and an international concern of the World Health Organization. It is highly contagious and, although the number of reported sick children is small at the moment, they are also vulnerable to infection. The importance of raising awareness and strengthening infection control measures can never be overemphasized.
